# Targeting Growth Factor and Cytokine Pathways to Treat Idiopathic Pulmonary Fibrosis

**DOI:** 10.3389/fphar.2022.918771

**Published:** 2022-06-03

**Authors:** Hongbo Ma, Shengming Liu, Shanrui Li, Yong Xia

**Affiliations:** ^1^ Department of Rehabilitation Medicine, West China Hospital, Sichuan University, Chengdu, China; ^2^ West China School of Pharmacy, Sichuan University, Chengdu, China; ^3^ Key Laboratory of Rehabilitation Medicine in Sichuan Province/Rehabilitation Medicine Research Institute, Chengdu, China

**Keywords:** cytokine, growth factor, signaling pathway, clinical trials, idiopathic pulmonary fibrosis, emerging pharmacotherapy

## Abstract

Idiopathic pulmonary fibrosis (IPF) is a chronic interstitial lung disease of unknown origin that usually results in death from secondary respiratory failure within 2–5 years of diagnosis. Recent studies have identified key roles of cytokine and growth factor pathways in the pathogenesis of IPF. Although there have been numerous clinical trials of drugs investigating their efficacy in the treatment of IPF, only Pirfenidone and Nintedanib have been approved by the FDA. However, they have some major limitations, such as insufficient efficacy, undesired side effects and poor pharmacokinetic properties. To give more insights into the discovery of potential targets for the treatment of IPF, this review provides an overview of cytokines, growth factors and their signaling pathways in IPF, which have important implications for fully exploiting the therapeutic potential of targeting cytokine and growth factor pathways. Advances in the field of cytokine and growth factor pathways will help slow disease progression, prolong life, and improve the quality of life for IPF patients in the future.

## 1 Introduction

Idiopathic pulmonary fibrosis (IPF) is a chronic, progressive fibrotic interstitial lung disease of unknown etiology that usually results in death from secondary respiratory failure within 2–5 years of diagnosis (Meltzer and Noble, 2008). It is a rare familial and sporadic disease. CT imaging of IPF usually shows a typical usual interstitial pneumonia (UIP) pattern, characterized by irregular reticular opacities with obligatory honeycombing, associated with traction bronchiectasis. IPF also exhibits histological features of UIP/IPF pattern characterized by dense fibrosis causing architecture remodeling with frequent honeycombing, patchy lung involvement by fibrosis, subpleural and/or paraseptal distribution, fibroblast foci at the edge of dense scars (Spagnolo et al., 2018; Baratella et al., 2021). With extensive basic and clinical research on the pathogenesis of IPF in recent years, some potential therapeutic targets have been discovered (Wang et al., 2021). A large number of these targets are growth factors, cytokines, and their signaling pathways, including TGF-β, CTGF, IL-13, CCL-2, leukotriene receptor, lipid proinflammatory mediators, and their downstream signaling. In addition, targeting pentraxin 2, galectin-3, oxidative stress, and B cell-mediated autoimmunity showed the potential to treat IPF. Lots of investigational drugs have entered clinical trials to test their efficacies in IPF therapy. However, there are only two currently approved IPF drugs, Pirfenidone and Nintedanib. They both slow the progression of IPF but are not able to reverse lung fibrosis (Chu et al., 2020). Lung transplantation is the only option for patients with end-stage IPF, but the donor organ shortages are an intractable problem worldwide, which means only a minority of patients have the opportunity to undergo lung transplantation (Spagnolo et al., 2018). In addition, lung transplantation is expensive, and the 10-year survival rate after surgery is only 33%–55% (Lederer and Martinez, 2018; Villavicencio et al., 2018). Therefore, there is still an urgent need to develop new drugs to treat IPF.

Although the pathophysiological mechanism of IPF remains unknown, significant progress in understanding the pathogenesis of IPF has been made in the last decade. The current paradigm assumes that recurrent alveolar epithelial cell injury and the crosstalk between dysregulated epithelial cells and mesenchymal, immune, endothelial cells can trigger abnormal wound healing responses and pulmonary fibrosis *via* multiple signaling pathways- (Mei et al., 2021; Moss et al., 2022). The pathogenesis of IPF is believed to be mediated by various cytokines, chemokines, and growth factors (Kelly et al., 2003). Cytokines and growth factors regulate the phenotypic switch of fibroblasts and alveolar epithelial cells (AECs), the recruitment and proliferation of mesenchymal cells, and the deposition and degradation of matrix through multiple mechanisms. Uncoordinated expression of several cytokines may be responsible for the severe matrix remodeling and epithelial-mesenchymal crosstalk in the lung microenvironment of IPF. Although the efficacy of previous anti-inflammatory treatments (e.g., TNF-α neutralization, immunosuppressants) and immunomodulatory treatments (e.g., interferon-γ) in clinical trials for the treatment of IPF has been unsatisfactory, targeting these pathways remains promising. Pirfenidone and Nintedanib, two small-molecule drugs that block multiple cytokine and growth factor signaling pathways, have been approved to slow the progression of pulmonary fibrosis. However, they have obvious defects, such as poor specificity caused by multiple targets and large doses, which lead to undesired side effects. Therefore, it is still necessary to explore the mechanism of the cytokine/growth factor pathway in IPF to find promising targets and develop targeted drugs.

Due to the pivotal role of the cytokine/growth factor pathway in the pathogenesis of IPF, this review comprehensively introduces the association of various growth factors, chemokines, interleukins, lipid proinflammatory mediators and their related signaling pathways with IPF. Related signaling pathways are also attractive therapeutic targets, including RTK and non-RTK pathways, Hedgehog pathway, Wnt pathway and Notch pathway, PI3K/Akt/mTOR pathway, MAPK pathway, and Hippo YAP/TAZ pathway. A large number of drugs, including many small molecules and biologics targeting cytokine/growth factor signaling pathways, have entered clinical trials to determine their efficacy against IPF, as described in this review. Taken together, the aim of this review is to provide an overview of cytokines, growth factors, and the related signaling pathways in IPF, thus providing a basis for the development of novel treatment options to alleviate or even reverse IPF.

## 2 The Pathological Process of Idiopathic Pulmonary Fibrosis

The complexity of the pathological process of pulmonary fibrosis lies in how crosstalk between epithelial-mesenchymal cells and multiple imbalanced cytokines contribute to the disease. First, alveolar epithelial cells (AECs) are damaged, and the continuity of the basement membrane is interrupted due to a variety of external stimuli, such as radiation, the microbiome, allergens, environmental particles, autoimmunity, antineoplastic drugs (Wilson and Wynn, 2009), and SARS-CoV2 (George et al., 2020). Then, many cytokines and growth factors are released by AECs to recruit and activate inflammatory cells and fibroblasts. Inflammatory cells and some coagulation factors [e.g., tissue factors (TF) and plasminogen activation inhibitors (PAI-1)] are jointly involved in the formation of wound clots (King et al., 2011b; Betensley et al., 2016). Activated AECs and endothelial cells participate in (myo)fibroblast migration, proliferation, and differentiation.

Under normal physiological conditions, the repair process of local lesions is controlled, but in IPF, epithelial-mesenchymal transition (EMT) and differentiation of fibroblasts into myofibroblasts occur, promoting the expansion of myofibroblast population which is the main source of extracellular matrix (ECM) (Yagihashi et al., 2016; Skibba et al., 2020). Notably, EMT may be an indirect mechanism of IPF, because it does not directly contribute to the expansion of the myofibroblast population *via* the epithelial-to-myofibroblasts transition (Rock et al., 2011; Salton et al., 2020). In contrast, EMT is indirectly involved in the pathological process of IPF through the paracrine of fibroblast activating factor (Hill et al., 2019). It has also been reported that EMT is the result of aberrant mechanical forces and signaling pathways in IPF (Qian et al., 2020; Saito et al., 2020; Salton et al., 2020; Su et al., 2020; Wu et al., 2020). Due to the presence of excess fibrin in the ECM, the lung elasticity of IPF patients decreases, which manifests as alveolar collapse and the cystic dilation of residual bronchioles/alveoli. This alveolar collapse leads to a decrease in the effective volume of the alveoli used for gas exchange. Moreover, matrix stiffness forms a positive feedback pathway through the mechanosensor transient receptor potential vanilloid 4 (TRPV4) and α6 integrin, which continuously aggravates pulmonary fibrosis (Rahaman et al., 2014; Chen et al., 2016), causing the loss of pulmonary function (respiratory ventilation) in IPF patients. In addition, excessive ECM encapsulates pulmonary capillaries, resulting in a decrease in the diffusion coefficients of oxygen and carbon dioxide and the loss of gas exchange function in the alveoli, eventually leading to the death of IPF patients from respiratory failure or related syndromes. If injury factors persist, AECs are continuously damaged, often manifesting as AEC death, an increase in the proportion of type II AECs (AT II), and impaired reepithelization (Liu et al., 2017). Therefore, different from normal repair, the repair processes involved in IPF are uncontrolled, continuous, and abnormal.

**FIGURE 1:**
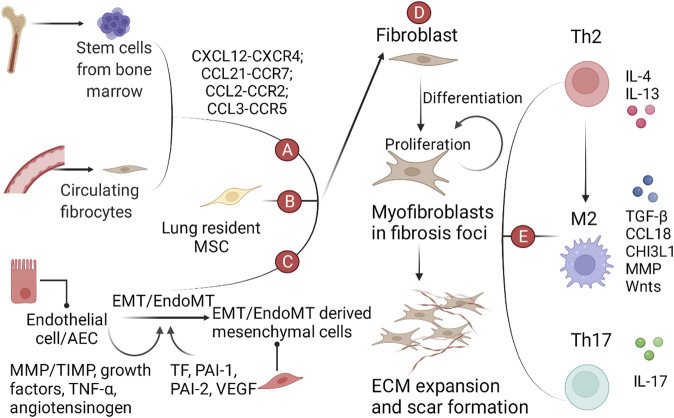
A schematic view of the role of various cells, growth factors and cytokines in IPF. **(A)** Bone marrow-derived mesenchymal progenitor cells and circulating fibrocytes in the peripheral circulation are recruited to the lung by the chemokine-chemokine receptor axis. **(B)** Lung resident MSCs. **(C)** EMT/EndoMT-derived mesenchymal cells. **(D)** Cells from the above three sources aggregate, proliferate, and differentiate into myofibroblasts in the lung, ultimately leading to ECM expansion and scarring. **(E)** Immune cells and cytokines involved in IPF.

A large number of cytokines and their signaling pathways are directly involved in the accumulation of fibroblasts in lung fibrosis foci (Figure 1). Fibroblasts are the culprits directly involved in pulmonary fibrosis, and their sources include stem cells recruited from the bone marrow, fibrocytes recruited from the peripheral circulation, mesenchymal stem cells (MSCs) residing in the lung, and EMT/EndoMT-derived interstitial cells. Plasma cells, etc. The CXCL12-CXCR4 axis (Phillips et al., 2004), CCL2-CCR2 axis (Chong et al., 2019), CCL3-CCR5 axis (Ishida et al., 2007; Besnard et al., 2013), and CCL21-CCR7 axis (Ziegenhagen et al., 1998; Habiel and Hogaboam, 2014) are directly involved in the recruitment of fibroblasts. EMT/EndoMT of epithelial and endothelial cells is affected by the coagulation cascade and angiogenesis-related cytokines (TF, PAI-1, and VEGF) and secretion released by AECs (TGF-β, growth factors, TNF-α, MMP/TIMP, and angiotensinogen), which are implicated in multiple signaling pathways (TGF-β, Wnt, SHH, Notch, and ER stress/UPR pathways) (King et al., 2011b; Selman and Pardo, 2014; Betensley et al., 2016).

Growth factors and cytokines are an integral part of the fibrotic microenvironment, which leads to differences in the phenotype of immune cells in the alveoli between patients with pulmonary fibrosis and healthy individuals. Th1/Th2 imbalance and M1-M2 polarization are hallmarks of pulmonary fibrosis. Th2 polarization is characterized by increased secretion of IL-4 and IL-13 and decreased secretion of IFN-γ. M2 polarization can be induced by the microenvironment shaped by Th2 polarization and promotes pulmonary fibrosis through the production of TGF-β, CCL18, chitinase 3-like 1 (CHI3L1), MMPs, and activation of the Wnt/β-catenin pathway (Shenderov et al., 2021). Th17 cells can promote fibroblast proliferation and ECM secretion by secreting IL-17 (Zhang et al., 2019a). In recent years, polymorphisms in immune-related genes have also been reported to be involved in the process of pulmonary fibrosis. For example, an increased risk or severity of IPF is associated with polymorphisms in the TLR3, Toll-interacting protein (TOLLIP), and interleukin-1 receptor antagonist (IL-1RA) genes (Whyte et al., 2000; Korthagen et al., 2012; Noth et al., 2013; O'Dwyer et al., 2013). In addition, activation of TLR2 and TLR9 has been reported to show profibrotic effects, whereas TLR3 has antifibrotic effects (Karampitsakos et al., 2017).

## 3 The Key Role of the TGF-β Signaling Pathway in Idiopathic Pulmonary Fibrosis

Transforming growth factor-β (TGF-β) is a potent profibrogenic cytokine that plays a central role in the development of pulmonary fibrosis by promoting fibroblast proliferation and phenotype modulation, stimulating the deposition of ECM, and participating in crosstalk with other cytokines and signaling pathways (Ong et al., 2021).

### 3.1 A Brief Introduction to TGF-β Signalling Pathway

**FIGURE 2:**
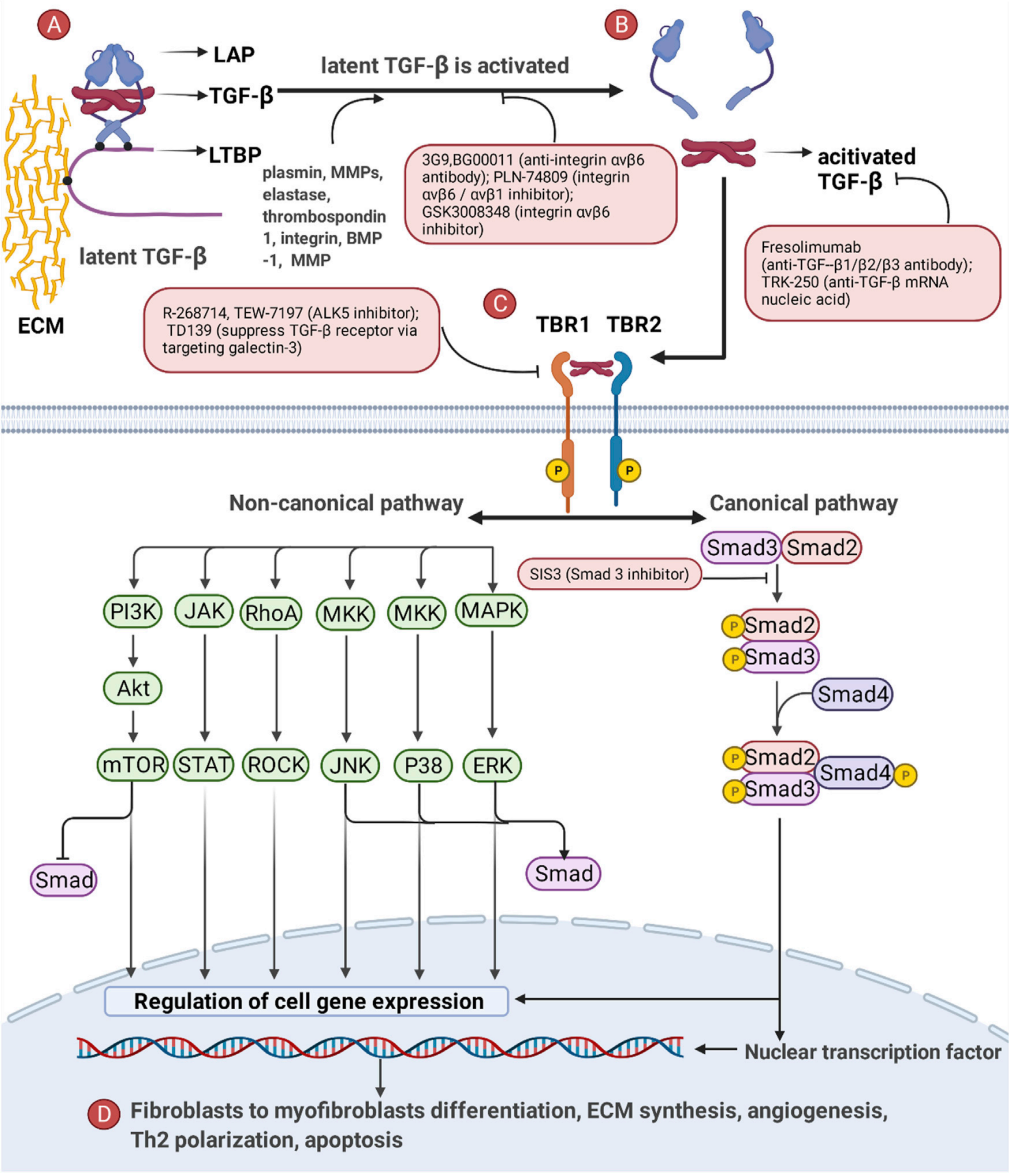
The critical role of the TGF-β pathway and drugs targeting the TGF-β pathway in IPF. **(A)** Latent TGF-β (TGF-β trapped in LAP) binds to ECM through LTBP. **(B)** TGF-β is detached from LAP and activated only in the presence of specific stimuli. **(C)** Activated TGF-β activates the downstream Smad-dependent pathway (canonical pathway) and Smad-independent pathway (non-canonical pathway) by combining with TBR. **(D)** The TGF-β pathway ultimately causes phenotypic reprogramming of AECs, fibroblasts and immune cells and influences fibroblast-to-myofibroblast differentiation, ECM synthesis, angiogenesis, Th2 polarization, and apoptosis.

The activation of TGF-β pathway includes five steps, including i) synthesis of TGF-β, ii) activation of latent TGF-β, iii) interaction between TGF-β and TGF-β receptor (TBR), iv) activation of classical and non-classical pathways, and v) regulation of nuclear transcription factors and cell phenotypes. A brief introduction to the TGF-β pathway is as follows and shown in Figure 2.

i) In the endoplasmic reticulum (ER), TGF-β precursors are assembled into dimers wrapped with latency-associated peptides (LAPs). TGF-β-LAPs bind to latent TGF-β binding protein (LTBP) and undergo intracellular proteolytic cleavage by the endopeptidase furin. Then, TGF-β-LAP-LTBPs are secreted into the extracellular medium and eventually bind to ECM (e.g., fibrillin and fibronectin) through LTBP. At this time, latent TGF-β cannot exert its biological function (Derynck and Zhang, 2003).
ii) Latent TGF-β is activated under special conditions [e.g., plasmin (Coutts et al., 2001), thrombospondin 1 (Murphy-Ullrich and Suto, 2018), elastase, integrin, BMP-1 and MMP 2 (Tzavlaki and Moustakas, 2020)] and binds to TGF-β receptor 2 (TBR2).
iii) TBR2 can phosphorylate TGF-β receptor 1 (TBR1) and activate the downstream classical and non-classical pathways.
iv) In classical pathways, Smad2/Smad3 activated by TBR1 binds to Smad4 to form Smad2/Smad3/Smad4, which enter the nucleus and participate in regulating transcription factors (Derynck and Zhang, 2003). Classical pathways also involve Smad7, ubiquitin, coactivator P100, PPAR-γ, Ski/SnoN, and other signal-regulating molecules (Tzavlaki and Moustakas, 2020). In non-classical pathways, TGF-β can activate many signaling pathways, such as the Ras-Raf-Mek1/2-ERK1/2 pathway, TAK1-MKK3/6-P38 pathway, TAK1-MKK4-JNK pathway, and PI3K-Akt-mTOR pathway (Derynck and Zhang, 2003; Ong et al., 2021), and participate in regulating nuclear transcription factors.
v) Some transcription factors of fibroblasts, alveolar epithelial cells, endothelial cells, and Th cells are changed, causing the phenotypic transformation of cells involved in pulmonary fibrosis, which eventually leads to the differentiation of myofibroblasts, the deposition of ECM, the abnormal function of AT II, and an imbalance of immune cells in IPF.

The TGF-β pathway is interrelated with the Wnt/β-catenin pathway, PI3K/Akt pathway, and pathways of other growth factors (Yan et al., 2014). Furthermore, in terms of the pathogenesis of IPF, TGF-β has been implicated in redox imbalance, mitochondrial dysfunction, EMT, MMP/TIMP imbalance, and fibrinolytic system imbalance (Chu et al., 2020).

### 3.2 Strategies to Treat IPF by Targeting TGF-β Pathway

Strategies for the treatment of IPF by targeting the TGF-β pathway include blocking TGF-β synthesis, preventing activation of latent TGF-β, neutralizing TGF-β ligands/receptors, and blocking canonical and non-canonical pathways (Saito et al., 2018; Hamanaka and Mutlu, 2021; Ong et al., 2021). These strategies are summarized in Figure 2.

Integrins mediate the mechanotransduction positive feedback to ECM stiffness in a TGF-β-dependent or TGF-β-independent manner, which is an important therapeutic target. TGF-β-dependent fibrosis is activated by integrins releasing TGF-β through tensile forces generated by actin-cytoskeleton interactions. In the TGF-β-independent pathway, after binding specific ECM ligands to the ectodomain of integrins, integrins bind to the cytoskeleton and various signaling proteins through their cytoplasmic tails, translating the mechanical force of cytoskeleton contraction and ECM stiffness into biochemical signals. Then, F-actin activates the downstream Rho/ROCK-YAP/TAZ signaling pathway *via* FAK phosphorylation, ultimately leading to fibroblast phenotype reprogramming.

There are corresponding preclinical candidate drugs for IPF that target different steps of the TGF-β pathways, including the synthesis, receptor binding, and downstream signal transduction (Ong et al., 2021), but no drug specifically for the TGF-β pathway has been approved due to the side effects of anti-TGF-β treatment (systemic autoimmune, cardiac valve problems, and carcinogenesis) (Henderson et al., 2020). Notably, to avoid systemic autoimmune disease induced by persistent systemic inhibition of TGF-β, it may be necessary to choose the correct dose or duration of treatment, coadminister anti-inflammatory drugs, selectively block TGF-β in targeted organs.

## 4 The Role and Underlying Mechanism of Cytokines and Growth Factors in Idiopathic Pulmonary Fibrosis

As described above, cytokines, growth factors, and related signaling pathways are intensively involved in the pathogenesis of IPF. Therefore, they might be promising targets to develop novel treatment options for IPF. In this section, we describe the roles of growth factors, chemokines, interleukins, lipid pro-inflammatory mediators, and their signaling pathways in IPF. Many drugs targeting these pathways are in development, and we summarize those that have entered clinical trials to treat patients with IPF. In recent years, therapeutics targeting these pathways have shown many limitations in clinical trials. Therefore, we propose possible approaches to overcome these limitations, aiming to provide insights into the development of therapies with fewer side effects and better efficacy.

### 4.1 Growth Factors

Growth factors can participate in the development and progression of IPF in TGF-β-dependent or TGF-β-independent ways. These growth factors comprise platelet-derived growth factor (PDGF), fibroblast growth factor (FGF), vascular endothelial growth factor (VEGF), epidermal growth factor (EGF), connective tissue growth factor (CTGF), and insulin-like growth factor (IGF). Due to the successful marketing of Nintedanib (an antagonist of PDGFR/VEGFR/FGFR), many studies have focused on growth factors and their corresponding receptors. However, there remains considerable controversy regarding the roles of many growth factors in promoting fibrosis and resisting fibrosis in IPF. One of the possible reasons for the controversy is that there are many subtypes of these growth factors and their receptors, and the functions of different subtypes differ. Although pharmacological analysis of these subtypes of growth factors is difficult, it is a vital step toward precise treatment and personalized treatment, which is of substantial significance. Next, we introduce the crucial growth factors PDGF, FGF, VEGF, EGF, and CTGF in detail.

#### 4.1.1 Platelet-Derived Growth Factor

Platelet-derived growth factor (PDGF) is a key growth factor that stimulates the proliferation and migration of fibroblasts. In a mouse model of bleomycin-induced IPF, RT–PCR (Maeda et al., 1996), PDGF antibody neutralization (Walsh et al., 1993), and Northern blotting (Zhuo et al., 2004) all demonstrated increased protein or mRNA expression of multiple subtypes of PDGF. However, the main subtypes of PDGF found in these experiments differed slightly, and the specific mechanism requires further research. A clinical study also observed that the high expression of PDGF was correlated with a low overall survival rate (Zhu et al., 2017).

Single strands encoded by the PDGF-A, B, C, and D genes can be combined in pairs to form five types of dimers, AA/BB/AB/CC/DD (Heldin and Westermark, 1999; Li and Eriksson, 2003; Günther et al., 2012). PDGFRs (PDGF receptors) are also homo/heterodimerically formed by combinations of single-chain PDGF-α/PDGF-β. After PDGF binds to the PDGF receptor, PDGFR dimerization can be induced; subsequently, the two PDGFRs after autophosphorylation can couple various downstream signal transduction pathways (Heldin and Westermark, 1999), such as Ras-mitogen-activated protein kinase (MAPK) through Grb2 and Shc adaptor proteins and PI3K and phospholipase C-γ (PLCγ) (Nishioka et al., 2013). PDGFR also participates in the migration and chemotaxis of fibroblasts through the integrin-FAK pathway. PDGF, as the main mitogen, can strongly promote the proliferation of fibroblasts and stimulate collagen synthesis (Heldin and Westermark, 1999). PDGF also participates in cell migration through Ca^2+^ influx and cytoskeleton rearrangement (Nishioka et al., 2013).

#### 4.1.2 Fibroblast Growth Factor

FGFs have been divided into seven subgroups encoded by 22 mammalian genomes. Among the 22 FGFs encoded by genomes, 4 FGFs are FGFR-independent, and the remaining 18 extracellular FGFs bind to 7 FGFR subtypes (FGFR1b, FGFR1c, FGFR2b, FGFR2c, FGFR3b, FGFR3c, and FGFRR4). An FGF can bind to multiple FGFRs. FGFs rely on heparan sulfate proteoglycan (HSPG) and Klotho-type coreceptors to improve their binding to FGFRs (Ornitz and Itoh, 2022). After FGF-FGFR binding, through the autophosphorylation of FGFRs, with the help of FGFR substrate 2 and PLCγ, signals are transferred to the RAS-ERK, PI3K/AKT, PKC, and JAK-STAT signaling cascades, which mediate the survival, proliferation, differentiation, or migration of cells (Inomata et al., 2015; Katoh, 2018).

Subtypes of FGFs have functional differences and play different roles in IPF (Yang et al., 2021). FGF1 (acid FGF or aFGF) has an anti-fibrotic function. The serum FGF1 level of patients with IPF was found to be higher than that of the control group (Shimbori et al., 2016). In rat models induced by TGF-β1, FGF1 could relieve IPF by inhibiting the TGF-β1 signaling pathway and promoting the proliferation of AECs (Shimbori et al., 2016). Further research found that “FGF1 + heparin treatment” could reverse EMT through the MAPK/ERK kinase pathway, leading to phosphorylation of ERK-1 and dephosphorylation of Smad2 (Ramos et al., 2010). FGF2 (basic FGF or bFGF) has a proliferative effect on lung fibroblasts (Hetzel et al., 2005; Khalil et al., 2005), and soluble FGFR2c significantly reduces TGF-β-induced IPF in mice (Ju et al., 2012). However, it has also been reported that FGF2 is antifibrotic in part through decreased collagen expression and fibroblast to myofibroblast differentiation (Koo et al., 2018). FGF9 and FGF18 promote the survival and migration of HLFs and inhibit myofibroblast differentiation *in vitro* (Joannes et al., 2016). More FGF9 was expressed in lung tissue myofibroblasts in patients with IPF than in healthy individuals, and it promoted epithelial cell growth and expansion of pulmonary interstitial through the Wnt7B/β-catenin signaling pathway (Yin et al., 2008). FGF10 plays an antifibrotic role *via* autocrine and paracrine signaling. During autocrine signaling, by activating peroxisome proliferator-activated receptor γ (PPAR γ), FGF10 blocks the lipofibroblast-to-myofibroblast transformation induced by TGF-β1 and promotes the transformation from myofibroblasts to lipofibroblasts. In addition, the FGF10 paracrine signal was considered crucial to the differentiation of alveolar epithelial progenitor cells during development and the maintenance of AT II in a steady state (Wu et al., 2018). FGF21 attenuates the TGF-β pathway *via* decreased oxidative stress in bleomycin-induced pulmonary fibrosis in mice (Zhang et al., 2018). Coadministration of FGF23 and its coreceptor α-Klotho led to a significant reduction in fibrosis and inflammation (Barnes et al., 2019).

In summary, FGF1, FGF10, FGF21, and FGF23 are anti-fibrotic, while FGF2, FGF9, and FGF18 exhibit contradictory functions.

#### 4.1.3 Vascular Endothelial Growth Factor

The VEGF family has seven members: VEGF-A, VEGF-B, VEGF-C, VEGF-D, VEGF-E, placental growth factor, and snake venom vascular endothelial growth factors (Inomata et al., 2015). Most studies have focused on the correlation between VEGF-A and IPF; thus, our description mainly focuses on VEGF-A. The most studied and dominant VEGF-A165 can be divided into VEGF-A165a and VEGF-A165b by the splice site. VEGF-A165a can promote angiogenesis, and VEGF-A165b can inhibit angiogenesis.

VEGF-A can bind to VEGFR1 (Flt-1), VEGFR2 (KDR or Flk1), and their coreceptors neuropilin-1 and neuropilin-2. Among them, VEGFR1 binds to circulating VEGF-α and reduces its bioavailability to VEGFR2. The coreceptors neuropilin-1 and neuropilin-2 assist in the signal transduction of VEGFR1 and VEGFR2. After binding to VEGFR2, VEGF-A165 can activate the downstream Akt pathway, Src signaling pathway, NCK and the p38 MAPK pathway, and integrin/FAK pathway (Fruttiger, 2008; Barratt et al., 2018). VEGF can stimulate the growth of alveolar epithelial type II cells and the production of alveolar surfactant, form new blood vessels, and help epithelial cells and endothelial cells resist apoptosis (Gerber et al., 1998a; Gerber et al., 1998b; Brown et al., 2001; Compernolle et al., 2002; Alavi et al., 2003; Mura et al., 2006; Roberts et al., 2007; Kuhn et al., 2010; Varet et al., 2010; Barratt et al., 2018).

Many contradictory effects of VEGF-A on IPF have been observed in animal models and clinical trials. The possible reasons for these differences are as follows: different animal models, different sampling sites, heterogeneity within and between individuals, and different VEGF-A subtypes caused by diverse splice sites. Different subtypes of VEGF-A may have mutually coordinated pathophysiological relationships. In *in vitro* experiments, VEGF-A165a has been proven to promote the proliferation of AT II and fibroblasts, increase the expansion of ECM, and play a role in promoting fibrosis, and VEGF-A165b could counteract this effect (Varet et al., 2010; Barratt et al., 2017). Thus, VEGF-A165b may be a compensatory protective mechanism.

#### 4.1.4 Epidermal Growth Factor

The EGF receptor (EGFR, also known as ErbB1 or HER1) belongs to the ErbB family receptor tyrosine kinases. EGFR has seven corresponding ligands: EGF, transforming growth factor-α (TGF-α), amphiregulin, betacellulin (BTC), epiregulin, epigen, and heparin-binding EGF-like growth factor (HB-EGF). After ligands bind to EGFR, the autophosphorylation of EGFR can activate the downstream MAPK, Akt, and JNK pathways and promote cell proliferation (Iwamoto and Mekada, 2012).

The relationship between the ErbB family (HER) and IPF remains unclear and thus requires further research. After blocking HER *in vivo*, studies have found that collagen deposition decreases and lung morphology improves, which indirectly indicates that the ErbB family plays a role in fibrosis (Rice et al., 1999; Faress et al., 2007). In addition, in patients with IPF, the mRNA level of EGFR was upregulated in proliferative alveolar epithelial cells around fibrosis, and the mRNA level of EGFR was positively correlated with the mRNA level of type I collagen and negatively correlated with the clinical prognosis (Tzouvelekis et al., 2013).

#### 4.1.5 Connective Tissue Growth Factor

CTGF, also known as CNN2, HCS24, or IGFBP8, belongs to the CNN family. CTGF can interact with a wide range of ECM components, but this also means that the biological action of CTGF is highly dependent on the local microenvironment. CTGF directly binds to other growth factors (e.g., TGF-β, BMPs, and VEGF), which influence signal emission/transduction (Choi, 2012). CTGF can be secreted by interstitial cells, such as proliferating AT II and activated fibroblasts (Pan et al., 2001). The peak of CTGF content mainly appears at the early stage of IPF, and the peak of CTGF appears earlier than the deposition of collagen in the lungs (Wang et al., 2011), which indicates that CTGF may be involved in the early-stage repair of lung tissue injury.

As an auxiliary regulator of TGF-β in IPF in the local microenvironment, CTGF can participate in abnormal tissue repair processes, such as ECM generation and the mobilization of fibroblasts, by assisting TGF-β (Wang et al., 2011). There was an interaction between TGF-β and CTGF in IPF animal models induced by TGF-β/bleomycin, and IPF could be alleviated by the CTGF antibody (FG-3019, pamrevlumab) (Wang et al., 2011). CTGF-deficient transgenic mice had the ability to resist IPF induced by bleomycin (Liu et al., 2011). Therefore, a general belief is that CTGF, as a fibrosis-promoting medium, is a possible target for the research and development of anti-IPF drugs. Pamrevlumab, a human recombinant mAb of CTGF, is the only antibody drug that has shown activity in a phase II clinical trial against IPF. In a recent randomized, placebo-controlled phase II trial for patients with IPF, pamrevlumab showed good safety and certain therapeutic effects, such as slowing the decline of pulmonary function and delaying the progression of fibrosis by HR-CT, which is a milestone of single-target therapy (Lipson et al., 2012; Raghu et al., 2016). The phase III clinical trial of pamrevlumab for IPF is at the recruitment stage (NCT04419558/NCT03955146).

### 4.2 Chemokines and Chemokine Receptors

Chemokines are small molecule proteins with chemotactic effects on specific cells, and there are four conserved cysteine residues. According to the differences in cysteine residues, chemokines have been divided into four main subfamilies: CXC, CC, CX3C, and XC. Although the role of some chemokines in IPF remains unknown, many studies have demonstrated that chemokines can promote fibrosis or resist fibrosis. It has been reported that pulmonary fibrosis in animal models can be attenuated by knocking out genes encoding chemokines or neutralizing chemokines with antibodies [CCL2-CCR2 (Moore et al., 2001; Murray et al., 2008; Phan et al., 2021), CCL11-CCR3 (Huaux et al., 2005), CCL17 (Agostini and Gurrieri, 2006), CCL21-CCR7 (Habiel and Hogaboam, 2014), CCL22 (Strieter, 2005), CXCL6 (Besnard et al., 2013), CXCL12 (Phillips et al., 2004), CXCL14 (Li et al., 2019), CX3CL1-CX3CR1 (Rivas-Fuentes et al., 2020), CCR5 (Ishida et al., 2007)]. The CXCL11-CXCR3 axis has anti-pulmonary fibrosis effects in a mouse model of bleomycin-induced pulmonary fibrosis (Strieter, 2005).

**FIGURE 3:**
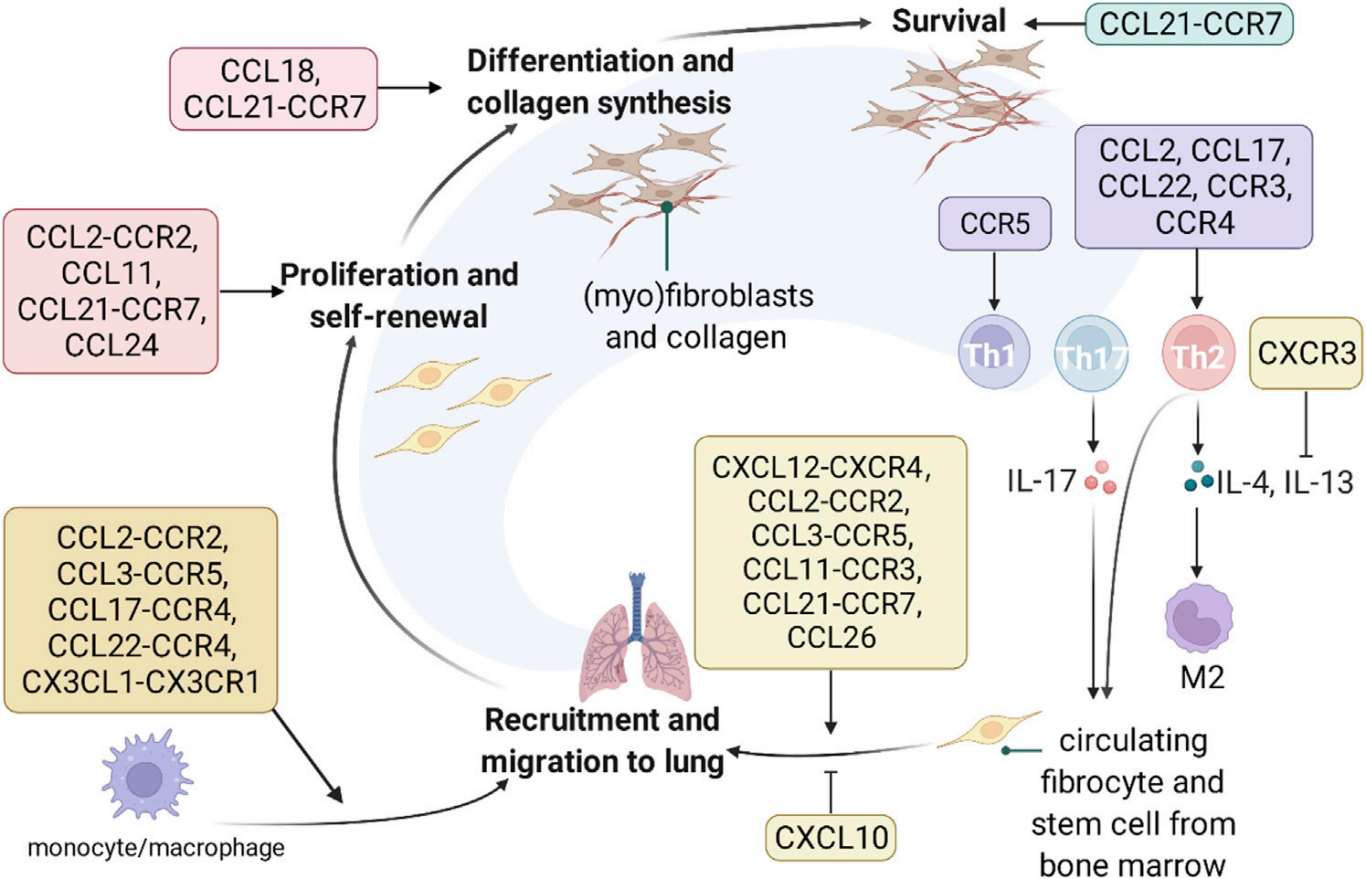
The role and underlying mechanisms of chemokines in IPF. Chemokines are involved in recruitment, proliferation, differentiation, collagen synthesis, survival of fibroblasts, regulation of type 1 and 2 immune balance, and recruitment of monocytes and macrophages.

Chemokines play a crucial role in the pathological process of IPF (Figure 3). CXCL12-CXCR4 (Phillips et al., 2004), CCL2-CCR2 (Chong et al., 2019) CCL3-CCR5 (Ishida et al., 2007; Besnard et al., 2013), CCL11-CCR3 (Puxeddu et al., 2006), CCL21-CCR7 (Ziegenhagen et al., 1998; Habiel and Hogaboam, 2014), CCL26 (Kohan et al., 2010) promote the migration of fibrocytes to the lung, whereas CXCL10 (Agostini and Gurrieri, 2006) inhibits the migration of fibrocytes to the lung. CCL2-CCR2 (Hambly et al., 2015), CCL11 (Puxeddu et al., 2006), CCL21-CCR7 (Ziegenhagen et al., 1998; Habiel and Hogaboam, 2014), CCL24 (Kohan et al., 2010) promote fibroblast proliferation. CCL18 (Hambly et al., 2015) and CCL21-CCR7 (Ziegenhagen et al., 1998; Habiel and Hogaboam, 2014) participate in the differentiation of fibroblasts to myofibroblasts and stimulate collagen synthesis. CCL21-CCR7 is involved in the survival of fibroblasts (Ziegenhagen et al., 1998; Habiel and Hogaboam, 2014). CCL2-CCR2 (Agostini and Gurrieri, 2006; Besnard et al., 2013; Hambly et al., 2015), CCL3-CCR5 (Ishida et al., 2007), CCL17-CCR4 (Karman et al., 2021), CCL22-CCR4 (Yogo et al., 2009), CX3CL1-CX3CR1 (Rivas-Fuentes et al., 2020) promote migration of monocytes and macrophages. CCL2 (Rose et al., 2003), CCL17 (Besnard et al., 2013), CCL22 (Besnard et al., 2013), CCR3 (Fulkerson et al., 2006), CCR4 (Yoshinouchi et al., 2007) are involved in type 2 immunity, whereas CCR5 (Loetscher et al., 1998) is involved in type 1 immunity. CXCR3 counteracts the profibrotic effect of IL-13 by assisting IL-13 receptor α2 gene expression (Pignatti et al., 2006; Yoshinouchi et al., 2007; Barnes et al., 2015). A phase II clinical trial of CNTO 888 (CCL2 mAb) failed to provide benefit to FVC in IPF patients (NCT00786201). Nonetheless, the role of chemokines in IPF remains to be further investigated.

### 4.3 Interleukins

Interleukins are a class of cytokines generated by lymphocytes, monocytes, and other non-monocytes. In addition to affecting fibrosis by modulating the Th1/Th2 balance, many studies have shown that interleukins can also directly affect fibroblasts and epithelial cells (Table 1). Thus, interleukins are expected to be developed as drugs and biomarkers for treating IPF.

**TABLE 1:** The role and underlying mechanism of interleukins in IPF.

Interleukins	Pro/anti-fibrotic	Mechanism of action	References
IL-1β	Pro-fibrotic	1) IL-1β-driven pulmonary fibrosis is dependent on IL-17A. 2) Gene polymorphism of IL-1β is associated with risk of IPF	Wilson et al. (2010); Korthagen et al. (2012)
IL-4	Pro-fibrotic	1) IL-4 can promote the synthesis of collagen I/III, fibronectin, and other ECM in fibroblasts. 2) IL-4 is a chemokine for fibroblasts. 3) IL-4-induced macrophage-derived IGF-I protects myofibroblasts from apoptosis	Wynes et al. (2004); Antoniou et al. (2007)
IL-6	Pro-fibrotic	1) The IL-6/STAT3/Smad3 axis has profibrotic effects. 2) IL-6 inhibits apoptosis of IPF-derived fibroblasts and promotes apoptosis of normal fibroblasts. 3) High levels of IL-6 and IL-8 are early features of AE-IPFs and are associated with worse outcome	Moodley et al. (2003); Papiris et al. (2018); Epstein Shochet et al. (2020)
IL-8	Pro-fibrotic	1) IL-8 promotes self-renewal, proliferation, and migration of mesenchymal progenitor cells in an autocrine manner. 2) IL-8 stimulates the recruitment and activation of macrophages in a paracrine manner	Yang et al. (2018)
IL-10	Anti-fibrotic	1) IL-10 has powerful anti-inflammatory effects. 2) IL-10 inhibits collagen I synthesis, although the antifibrotic effect of IL-10 is controversial	Arai et al. (2000); Barbarin et al. (2005); Nakagome et al. (2006); García-Prieto et al. (2010); Li et al. (2011)
IL-11	Pro-fibrotic	IL-11 promotes fibrosis *via* JAK/STAT pathway, Ras/Raf/MEK/ERK1/2 pathway, and PI3K/Akt/mTOR pathway	Ng et al. (2019); Kortekaas et al. (2021)
IL-13	Pro-fibrotic	IL-13 stimulates fibroblast proliferation, and induces TGF-β, PDGF, CTGF, collagen I, and fibronectin production	Murray et al. (2008); Passalacqua et al. (2017)
IL-17	Pro-fibrotic	1) IL-17A promotes cell proliferation, ECM deposition, and myofibroblast differentiation through NF-κb and JAK2 signaling. 2) IL-17B is also involved in dysbiosis of lung microbiota. IL-17 cooperates with TGF-β1-mediated Smad2/3 and ERK1/2 to induce EMT in human pulmonary alveolar epithelial cells	Wilson et al. (2010); Wang et al. (2017); Zhang et al. (2019a); Yang et al. (2019)
IL-18	Pro-fibrotic	IL-18 promotes senescence and SASP in pulmonary fibroblasts by blocking the Klotho pathway	Zhang et al. (2019b)
IL-22	Anti-fibrotic	IL-22 inhibits TGF-β-induced signaling pathways and reduces EMT and myofibroblast differentiation	Gu et al. (2021)
IL-24	Pro-fibrotic	IL-24 cooperates with IL-4 to promote macrophage M2 polarization	Rao et al. (2021)
IL-25	Pro-fibrotic	1) IL-25/IL-33/TSLP^+^ AECs-IL-25R/IL-33R/TSLPR^+^ (myo)fibroblasts axis is involved in epithelial-mesenchymal crosstalk. 2) Autocrine IL-25/IL-33/TSLP (thymic stromal lymphopoietin) from alveolar epithelial cells can cause damage and phenotypic changes in alveolar epithelial cells	Xu et al. (2020)
IL-31	Pro-fibrotic	1) IL-31 regulates the transcription of ECM and AECs-related genes. 2) IL-31 can cause collagen deposition and decreased lung function	Yombo et al. (2021)
IL-37	Anti-fibrotic	1) IL-37 resulted in enhanced autophagy and attenuated TGF-β1 of IPF fibroblasts. 2) IL-37 inhibits oxidative stress-induced death of AECs	Kim et al. (2019)

However, clinical trials of IPF treatments targeting IL-4 and IL-13 have not gone well. Several drugs targeting IL-13 have entered clinical trials for IPF, but none of them have shown protective effects on lung function. Although clinical trials of IPF antibody drugs have mostly failed in phase II, there is still hope for interleukin therapy. Inhibition of IL-11 blocks TGF-β1, PDGF, FGF2, IL-13, OSM (Oncostatin M), and endothelin 1-mediated fibroblast activation (Ng et al., 2019).

Notably, interleukin supplementation alone may not reverse the profibrotic phenotype of cells, and the regulation of cell–cell interactions or phenotypic transformation may be more promising. In addition, anti-inflammatory interleukins should be used with caution in clinical trials, and attention should be given to their side effects on patient immune function. In addition, rational design of interleukin dosage forms is very important because the concentration of the drug in the lungs can explain some of the differences in the study results.

### 4.4 Lipid Proinflammatory Mediators

Various lipids and their metabolic derivatives play vital roles in IPF. Although glucocorticoids (PLA2 inhibitor) have not shown positive effects in the treatment of IPF in clinical trials, these findings do not indicate that all lipid metabolism pathways have no significance as therapeutic targets of IPF. In contrast, metabolomic studies on fibroblasts have received increasing attention in recent years.

LT and PG are implicated in the pathogenesis of IPF. In arachidonic acid metabolic pathways, phospholipids produce arachidonic acid under the catalysis of phospholipase A2 (PLA2). Then, arachidonic acid produced leukotriene (LT) and prostaglandin (PG) under the catalysis of 5-lipoxygenase (5-LO) and cyclooxygenase (COX), respectively. Among them, PGF2a and LTs promote fibrosis, and PGE2 resists fibrosis (Suryadevara et al., 2020). A phase II trial (NCT02503657) on the safety and tolerability of Tipelukast/MN-001 (LT receptor inhibitor, 5-LO inhibitor and PDE inhibitor) in patients with IPF is ongoing.

The SPHK1/S1P/S1PR axis is involved in pulmonary fibrosis. In sphingolipid metabolism, sphingosine kinase 1 (SPHK1) phosphorylates sphingosine to produce sphingosine-1-phosphate (S1P). Then, the binding of S1P to the S1P receptor can lead to mitochondrial reactive oxygen species (mtROS) and promote YAP1 entry into cell nuclei, affecting the differentiation of myofibroblasts and matrix remodeling (Huang et al., 2020). Targeting the SPHK1/S1P/S1PR axis, PF543 (SPHK1 inhibitor), Mito TEMPO (mitochondria-targeted superoxide dismutase, which can reduce mtROS) and verteporfin (YAP inhibitor) have been reported, but these drugs have not entered clinical trials on IPF treatment.

The ATX/LPA/LPAR axis plays a potent role in pulmonary fibrosis. Lysophosphatidic acid (LPA) has been proven to activate G protein-mediated signal transduction pathways by binding to its receptors (LPAR1 and LPAR2), which leads to different reactions from lung cells, including the promotion of the apoptosis of epithelial cells, regulation of endothelial permeability, activation of αvβ6 integrin-mediated TGF-β signaling, secretion of IL-8, and recruitment and survival of fibroblasts (Tager et al., 2008; Ninou et al., 2018). LPA can promote the apoptosis of epithelial cells (Suryadevara et al., 2020). LPA is produced by many metabolic pathways *in vivo*, among which ATX/LPA/LPAR is the main pathway. Phosphatidylcholine (PC) generates LPC (lysophosphatidylcholine) under the action of phospholipase A1/phospholipase A2 (PLA1/PLA2), and LPC is hydrolyzed under the action of autotaxin (ATX)/lysoPLD to generate LPA. LPA exerts biological effects by binding to LPAR (Suryadevara et al., 2020). Drugs targeting different positions of the ATX/LPA/LPAR axis have entered clinical trials. The ATX inhibitors BBT-877 and GLPG1690 entered phase I clinical trials (completed) and phase III clinical trials (terminated) on their anti-IPF treatment, respectively. The LPA1 receptor antagonists BMS-986278 (recruiting) and BMS-986020 (completed) have also entered phase II clinical trials.

## 5 Growth Factor and Cytokine Signaling Cascades in Idiopathic Pulmonary Fibrosis

### 5.1 Receptor-Type Tyrosine Kinases and Non-Receptor-Type Tyrosine Kinases Signaling Cascades

**FIGURE 4:**
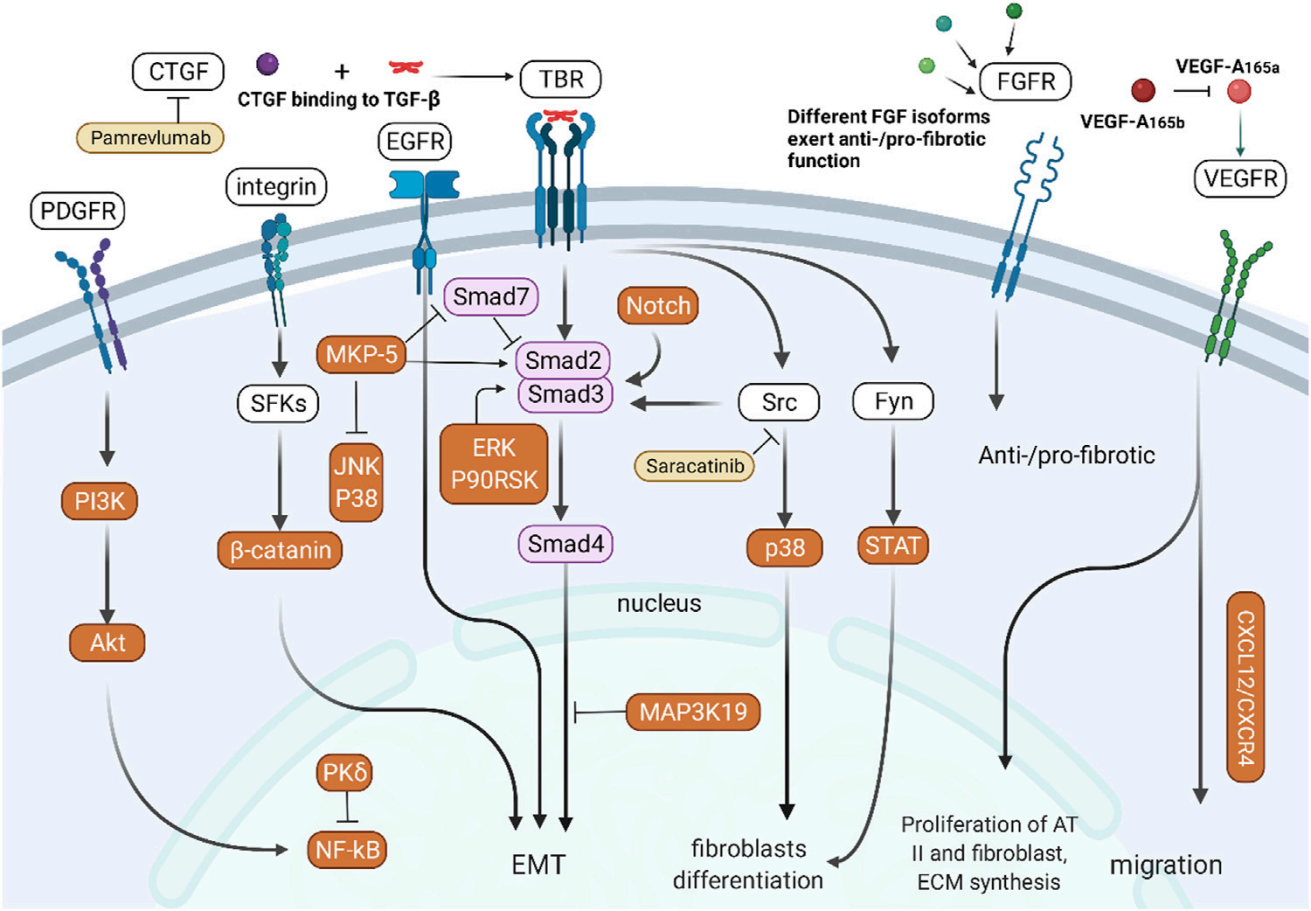
Crosstalk between RTK/non-RTK and other signaling pathways. PDGFR activates NF-κB signaling through the PI3K/Akt pathway. Integrins participate in EMT of epithelial cells through SFK/β-catenin-mediated signaling. TGF-β/Smad signaling interacts with the CTGF, Src, Fyn, and Notch pathways to participate in EMT in epithelial cells. Different subtypes of FGF and VEGF have different pro- or antifibrotic effects.

Due to the central role of growth factors in IPF, the signaling cascade of growth factor receptors is an indispensable topic. Growth factor receptors (GFRs) are receptor-type tyrosine kinases (RTKs), and their counterparts are non-receptor-type tyrosine kinases (non-RTKs) free in the cytoplasm. Src family kinases (SFKs) are a non-RTK family with eleven members, among which Src, Yes and Fyn are ubiquitously reported (Li et al., 2020). RTK and non-RTK signaling crosstalk with numerous other pathways and contribute to fibrosis *via* phenotype modulation of fibroblasts and AECs (Figure 4).

The combination of TGF-β and TBR can not only initiate the TGF-β/Smad signaling pathway but also indirectly participate in the differentiation of fibroblasts by activating Src/p38 (Pechkovsky et al., 2008) and Fyn/STAT (Xu et al., 2019). Integrins can activate SFKs to activate β-catenin and participate in EMT of epithelial cells (Ulsamer et al., 2012). CTGF participates in abnormal tissue repair by assisting TGF-β. The Notch pathway can also promote TGF-β-mediated fibroblast differentiation through activation of Smads (Aoyagi-Ikeda et al., 2011). MAP3K19 regulates nucleocytoplasmic shuttling of the activated R-Smads, which promotes TGF-β-mediated fibrosis (Boehme et al., 2016). MKP-5, a tyrosine phosphatase negatively regulating the p38 and JNK pathways, inhibits Smad7 activity but promotes Smad3 phosphorylation and the expression of fibrogenic genes (Xylourgidis et al., 2019).

PDGFR activates NF-κB *via* the Src/PI3K/Akt pathway (Cheng et al., 2014). PKCδ attenuates pulmonary fibrosis by enhancing the stability and activity of A20, an inhibitory protein of NF-κB signaling. The PDGFR/mTOR signaling pathway is associated with PINK1/PARP2 dysregulation-induced mitophagy deficiency, leading to myofibroblast differentiation and proliferation (Tsubouchi et al., 2018). Different isoforms of FGF participate in the process of fibrosis through FGFR; for example, FGF1 (Ramos et al., 2010) and FGF10 (Cheng et al., 2014) are anti-fibrotic, while FGF2 is pro-fibrotic (Ju et al., 2012). VEGF-A165a has been proven to promote the proliferation of AT II cells and fibroblasts and ECM expansion, and VEGF-A165b could counteract this effect (Varet et al., 2010; Barratt et al., 2017). VEGFR1 induces pulmonary fibrosis by promoting the migration of VEGFR1+ cells, which is dependent on the SDF-1/CXCR4 axis (Amano et al., 2021). In addition to the above GFR, Gas6/TAM receptor-type tyrosine kinases have also been reported to promote pulmonary fibrosis (Espindola et al., 2018).

### 5.2 Developmental Pathways: Hedgehog, Wnt, and Notch

**FIGURE 5:**
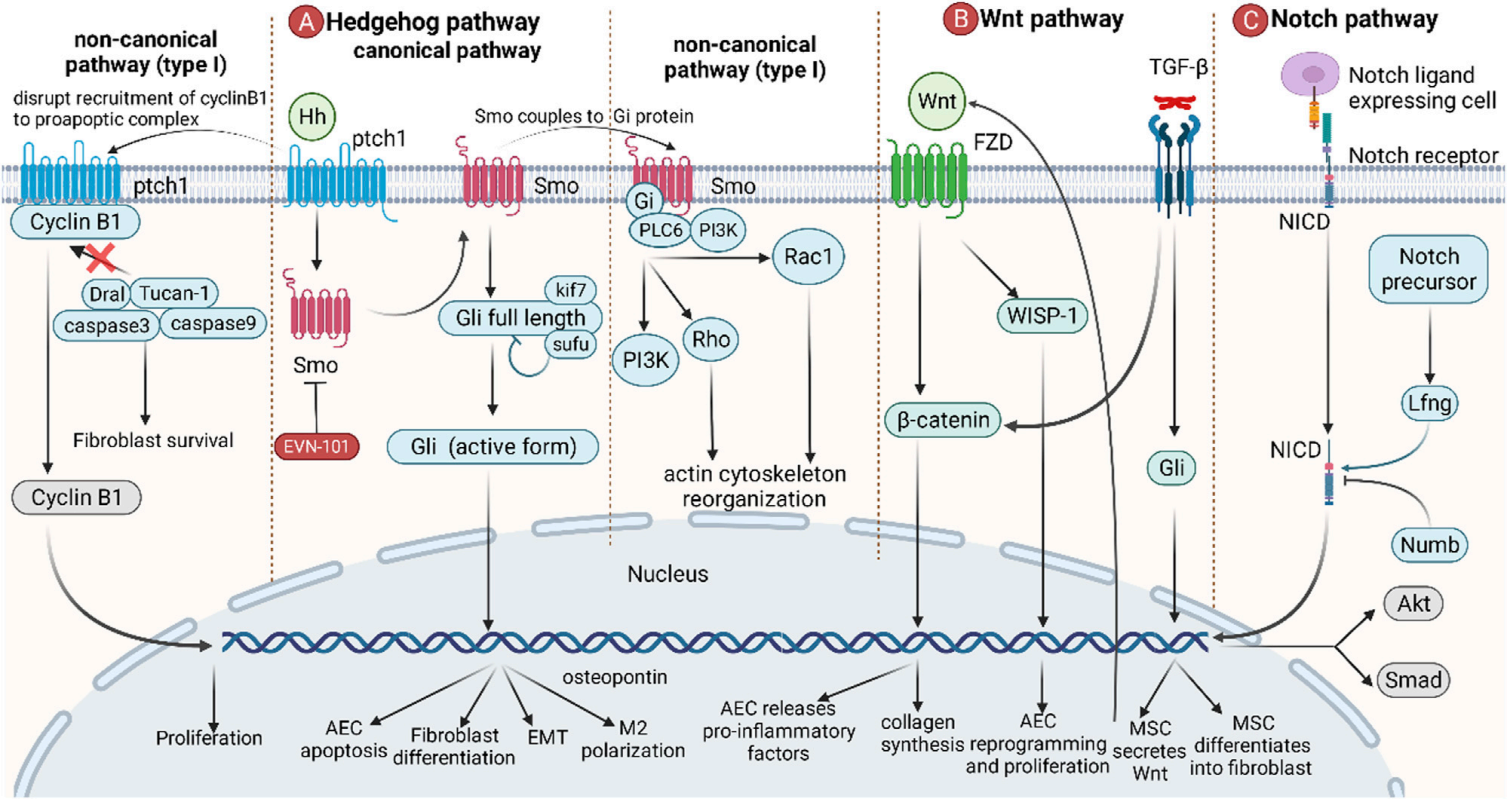
The role of developmental pathways in IPF. **(A)** Canonical hedgehog pathways start from the binding of Hh to ptch1, and Smo then migrates to the plasma membrane, releasing and activating full-length Gli, which leads to AEC apoptosis, fibroblast differentiation, EMT, and M2 polarization. In non-canonical hedgehog pathways (type I), binding of Hh to ptch1 blocks the recruitment of the pro-apoptotic complex by cyclin B1, resulting in anti-apoptosis and promotion of cell proliferation. In non-canonical hedgehog pathways (type II), Smo is coupled to Gi protein, activating downstream PI3K, Rho, and Rac1, resulting in an increase in intracellular calcium concentration and a rearrangement of the cytoskeleton. **(B)** Wnt promotes the accumulation of β-catenin by binding to FZD, thereby stimulating AECs to release proinflammatory factors and promote collagen synthesis in fibroblasts. Wnt also directly induces AEC reprogramming and proliferation *via* WISP-1. TGF-β can induce MSCs to secrete Wnt and promote MSC differentiation. **(C)** The Notch ligand binds to the Notch receptor, causing the Notch intracellular domain (NICD) of the Notch receptor to break and enter the nucleus, leading to gene transcription. Lfng positively regulates this process, while Numb negatively regulates this process. Wnt is also involved in the regulation of Akt and Smad pathways.

Developmental pathways play an important role in the developmental stages of the lung. In adults, some developmental pathways enter a dormant state but are activated again when abnormal lung damage occurs. These signaling pathways include the TGF-β, FGF, Hedgehog, Wnt, and Notch signaling pathways (Chanda et al., 2019) (Figure 5). Among them, TGF-β and FGF have been mentioned in the previous section and will not be repeated here.

The Hedgehog pathway starts with the Hedgehog ligand (Hh) and its receptor ptch1 and then activates downstream pathways in a Gli transcription factor-dependent (classical) or independent (non-classical) manner. In the canonical pathway, the binding of Hh to ptch1 relieves the inhibitory effect of ptch1 on Smo. Then, Smo is transferred to the plasma membrane and participates in the regulation of genes in the nucleus by activating full-length Gli to become Gli1/2/3 (active form), thereby inducing AEC apoptosis, fibroblast differentiation, EMT, and M2 polarization (Effendi and Nagano, 2021). In the non-canonical pathway (Type I), the binding of Hh to ptch1 prevents cyclin B1 from recruiting proapoptotic complexes, thereby failing to activate caspase-mediated apoptosis. After entering the nucleus, cyclin B1 can also regulate cell cycle progression by promoting cell proliferation (Robbins et al., 2012; Effendi and Nagano, 2021). In the non-canonical pathway (Type II), the coupling of Smo to Gi protein activates downstream PI3K, Rho, and Rac1, leading to an increase in intracellular calcium concentration and cytoskeleton rearrangement (Robbins et al., 2012; Effendi and Nagano, 2021).

The Wnt signaling pathway is an important signaling pathway in response to postnatal injury and regeneration. Binding of Wnt3a to Frizzled (FZD), followed by β-catenin accumulation, induces AECs to release proinflammatory factors (IL-6, IL-1β) and promotes fibroblast collagen synthesis. In addition, Wnt-inducible signaling protein-1 (WISP-1) is also involved in AEC reprogramming and proliferation (Königshoff et al., 2009). TGF-β promotes the differentiation of mesenchymal stem cells (MSCs) into fibroblasts by activating Gli to secrete Wnt5a, Wnt10a, and Wnt7b, thereby initiating the Wnt pathway (Chen et al., 2018; Martin-Medina et al., 2018). Since Wnt signaling is associated with a large number of normal physiological functions, there are currently no anti-IPF drugs targeting this pathway.

The Notch pathway is a highly conserved signaling pathway that mediates short-range signaling in neighboring cells. The Notch ligand-expressing cell binds to the Notch receptor, causing the Notch intracellular domain (NICD) of the Notch receptor to break and enter the nucleus to lead to gene transcription. Lfng positively regulates this process, while Numb negatively regulates this process (Kiyokawa and Morimoto, 2020). Notch is also involved in the TGF-β/Smad3-mediated transformation of fibroblasts to myofibroblasts (Aoyagi-Ikeda et al., 2011). Notch also induces phosphorylation of Akt by stimulating the expression of insulin-like growth factor-1R (IGF1R), which in turn promotes pulmonary fibrosis by hypoxia-inducible factor-1 (HIF-1) (Eliasz et al., 2010).

### 5.3 Other Critical Signaling Cascades in Idiopathic Pulmonary Fibrosis: JAK/STAT, PI3K/Akt/mTOR, MAPK, and Hippo-YAP/TAZ

**FIGURE 6:**
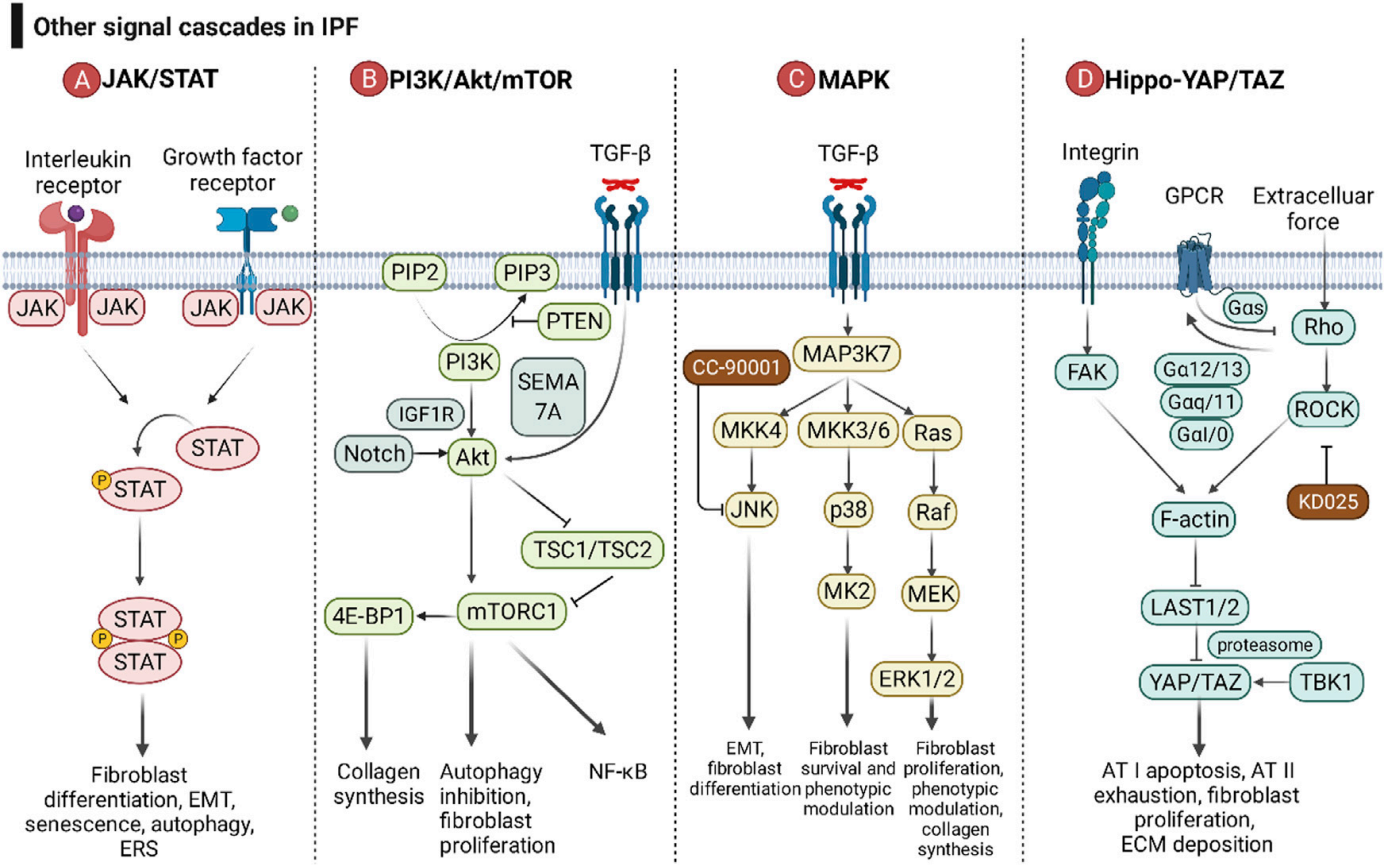
The roles of the JAK/STAT pathway, PI3K/Akt/mTOR pathway, MAPK pathway, and Hippo-YAP/TAZ pathway in IPF. **(A)** After interleukins and growth factors bind to the corresponding receptors, they can activate the JAKs coupled to the receptors, and then JAKs can phosphorylate STAT. Homodimerized STAT enters the nucleus and affects gene transcription. **(B)** Growth factors and cytokines can activate PI3K by binding to receptor-type tyrosine kinases, and then PI3K catalyzes the phosphorylation of PIP2 to PIP3. PIP3 can activate Akt and its downstream mTOR complex (mTORC). PTEN is a negative regulator of the PI3K/Akt/mTOR pathway. TGF-β and Notch are positive regulators of Akt. **(C)** The MAPK pathway consists of three downstream cascades, the JNK, p38, and ERK pathways. JNK and p38 signaling are activated through a pattern of MAP3K-MAP2K-MAPK signaling cascades, while ERK is activated through the MAP3K-Ras-Raf-MEK-ERK cascade. MK2 is a downstream signaling molecule of p38. Growth factors can activate ERK5, and ERK5 and p90RSK can acetylate Smad, thereby promoting the TGF-β/Smad pathway. **(D)** The Hippo-YAP/TAZ pathway is involved in pulmonary fibrosis through the integrin pathway, G protein-coupled receptor pathway, and mechanotransduction pathway. ECM force initiates the Hippo-YAP/TAZ pathway with activation of FAK and Rho/ROCK. FAK and Rho/ROCK relieve the inhibition of the transcription factor YAP/TAZ by large tumor suppressor kinase 1 and 2 (LAST1/2) by promoting the growth, stability, and contractibility of F-actin. Different GPCR signaling pathways regulate LAST1/2 activity through the Rho/ROCK/F-actin pathway.

In recent years, research on the molecular mechanism of fibrosis has become increasingly in depth, and the JAK/STAT, PI3K/Akt/mTOR, MAPK, and Hippo-YAP/TAZ signaling cascades have received extensive attention (Figure 6). Drugs targeting these signaling pathways are already in clinical trials.

Cells respond to inflammatory factors rapidly through JAK/STAT signaling. After interleukins (IL-4, IL-6, IL-11, IL-13, and IL-31) and growth factors (TGF-β, PDGF, VEGF, EGF, and FGF) bind to the corresponding receptors, they can activate and interact with the receptors. Receptor-coupled JAKs (JAK1, JAK2, JAK3, and TYK2) phosphorylate STATs (STAT1, STAT2, STAT3, STAT5, and STAT6) (Montero et al., 2021). Homodimerized STAT enters the nucleus to affect gene transcription, leading to fibroblast differentiation, EMT, senescence, autophagy, and ERS (Montero et al., 2021). Despite numerous isoforms of JAK and STAT, JAK2/STAT3 plays a dominant role in pulmonary fibrosis (Milara et al., 2018). Jaktinib dihydrochloride monohydrate and CC-90001, as JAK1/2 inhibitors, have entered phase II clinical trials (NCT04312594 and NCT03142191, respectively). Since different subtypes of STAT share downstream pathways, blocking only one subtype of STAT may lead to ineffective treatment due to the compensatory effect of other subtypes of STAT. Therefore, no therapeutic drugs targeting STAT have entered clinical trials.

PI3K/Akt/mTOR signaling is involved in autophagy and has caused a huge hit in the field of tumor therapy. A genome-wide association study revealed an association of mTOR with susceptibility to fibrosis (Allen et al., 2020). Growth factors and cytokines activate PI3K by binding to receptor-type tyrosine kinases, which in turn catalyze the phosphorylation of PIP2 to PIP3. PIP3 activates Akt and its downstream mTOR complex (mTORC), which promotes collagen synthesis, proliferation in fibroblasts, and EMT in epithelial cells (Lawrence and Nho, 2018; Fang et al., 2020). PTEN can inhibit the phosphorylation of PIP2, thereby inhibiting the signaling pathway downstream of PI3K and reducing the senescence of AECs (Qiu et al., 2019). Akt relieves the inhibition of mTORC1 by TSC1/TSC2 by phosphorylating TSC2 (Huang and Manning, 2009). mTOR also intersects with NF-κB signaling (Guo et al., 2013). TGF-β signaling can activate Akt in a SEMA 7A-dependent manner, which in turn activates downstream mTOR signaling and promotes lung fibrosis (Reilkoff et al., 2013). mTOR can activate collagen synthesis *via* 4E-BP1 (Woodcock et al., 2019). RPS6KB2 (ribosomal protein S6 kinase B 2) is reported to be involved in the process of aging and IPF due to its activation by growth factors and regulation of the protein kinase mTOR signaling pathway (Hao et al., 2017).

Mitogen-activated protein kinase (MAPK) mainly consists of three downstream cascades, the JNK, p38 and ERK pathways. JNK/p38 is activated *via* a MAP3K-MAP2K-MAPK signaling cascade, whereas ERK is activated *via* a MAP3K-Ras-Raf-MEK-ERK cascade (Woodcock et al., 2019). JNK and p38 signaling participates in apoptosis, necroptosis, and EMT of AEC; fibroblast-to-myofibroblast differentiation; and maintenance of the myofibroblast phenotype (Kasuya et al., 2021). MK2, downstream of the p38 pathway, has a role in fibroblast invasion and fibrosis (Liang et al., 2019). Growth factors can activate ERK5, and then ERK5 and p90RSK can acetylate Smad, thereby promoting the TGF-β/Smad pathway (Kim et al., 2020). The ERK1/2-calpain pathway has been reported to be associated with pulmonary fibrosis *in vivo* and *in vitro* (Zou et al., 2020).

The Hippo-YAP/TAZ pathway is involved in pulmonary fibrosis through the integrin pathway, G protein-coupled receptor pathway, and mechanotransduction pathway. Integrins promote the growth, stability, and contractibility of F-actin by activating FAK, thereby relieving the inhibitory effect of large tumor suppressor kinase 1 and 2 (LAST1/2) on YAP/TAZ. After entering the nucleus, YAP/TAZ regulates AT I apoptosis and AT II exhaustion, fibroblast proliferation and ECM expansion (Sun et al., 2021). The mechanical force of ECM can also activate F-actin *via* Rho/ROCK signaling and promote pulmonary fibrosis (Zhou et al., 2013). Different GPCR signaling pathways (Gαs, Gα12/13, Gαq, and Gαi/o) regulate LAST1/2 activity through the Rho/ROCK/F-actin pathway (Haak et al., 2020). Tank binding kinase 1 (TBK1) activates YAP/TAZ in a proteasomal machinery-dependent but LAST-independent manner (Aravamudhan et al., 2020).

## 6 Advances in the Development of Anti-IPF Drugs Targeting Cytokine and Growth Factor Pathways

### 6.1 Pirfenidone and Nintedanib, Two FDA-Approved Drugs for Patients With Idiopathic Pulmonary Fibrosis

Antifibrotic therapy provides survival benefit and protection against all-cause and respiratory-related hospitalization for IPF patients (Mooney et al., 2021). FDA approved antifibrotic drugs for treating IPF include Pirfenidone and Nintedanib, and their clinical efficacy is similar though there are no high-quality clinical trials directly comparing the efficacy of the them (Marijic et al., 2021). Regarding their mechanism of action, both of them target multiple growth factor and cytokine-related signaling pathways. Now that the pathogenesis of IPF remains unclear, targeting multiple pathways may be an effective treatment strategy for IPF. In addition, since the mechanisms of Pirfenidone and Nintedanib are not the same, a combination treatment may have synergistic effects. The combination of Pirfenidone and Nintedanib has been shown to be well tolerated and safe, which encourages further research into combination therapy (Flaherty et al., 2018; Vancheri et al., 2018). How to relieve the side effects caused by the off-target effects of Pirfenidone and Nintedanib is also an important topic.

#### 6.1.1 Pirfenidone

Pirfenidone is a pyridine compound approved for marketing in Japan in 2008. It was originally developed as an anti-inflammatory drug. Interestingly, studies in animal models demonstrated that it had the effect of resisting fibrosis of various organs, and it was later approved for IPF treatment. The anti-fibrotic mechanism of Pirfenidone has not been fully elucidated. Published studies indicated that it could inhibit fibrosis mainly by blocking TNF-α and TGF-β/Smad pathways (Oku et al., 2002; Schaefer et al., 2011; Conte et al., 2014).

Three large-scaled RCTs demonstrated that Pirfenidone could delay FVC decline, improve progression-free survival, increase exercise tolerance, and reduce all-cause or IPF related mortality (Taniguchi et al., 2010; Noble et al., 2011; Noble et al., 2012; King et al., 2014). Recently, a systematic review and meta-analysis of RCTs also indicated that Pirfenidone treatment was associated with a longer progression-free-survival and a lower incidence of acute exacerbation (Wu et al., 2021). In the real-world practice, Pirfenidone also provided beneficial effects on survival and pulmonary function decline (Lee et al., 2021). Moreover, A *post hoc* analysis of ASCEND and CAPACITY program suggested a clinically relevant benefit of Pirfenidone in IPF patients with more advanced lung function impairment (Nathan et al., 2019).

However, the use of Pirfenidone has some limitations, such as a short half-life (2.5 h) and a high daily dose (2,403 mg/day). Although generally well tolerated, a minority of patients discontinued treatment due to gastrointestinal and skin-related adverse events. These side effects can be mitigated or prevented by taking it with/after meals, avoiding Sun exposure, wearing protective clothing, and using broad-spectrum sunscreens (Costabel et al., 2014). In addition, caution is recommended prior to Pirfenidone use in IPF patients with severe hepatic and renal insufficiency.

#### 6.1.2 Nintedanib

Nintedanib is a small molecule tyrosinase inhibitor approved for marketing in 2014. Initially, researchers intended to screen drugs that selectively inhibit VEGFR-2 for anticancer treatment. However, in subsequent research, Nintedanib had good antifibrotic and anti-inflammatory effects and was approved for IPF treatment. Nintedanib mainly inhibits tyrosine kinase receptors, such as PDGFR, FGFR, and VEGFR, and non-receptor tyrosine kinases, such as Src and Flt-3 (Hilberg et al., 2008).

The INPULSIS-1 and INPULSIS-2 programs demonstrated that Nintedanib could delay FVC decline and reduce the risk of disease progression (Richeldi et al., 2014; Keating, 2015). However, the parameter of time to first AE and HRQL (Health-related Quality of Life) was inconsistent between INPULSIS-1 and INPULSIS-2 (Keating, 2015). Interestingly, despite similar efficacy of Pirfenidone and Nintedanib, there was a higher rate of discontinuation of Nintedanib due to adverse effects (Takehara et al., 2022). Gastrointestinal adverse events (diarrhea, nausea, and vomiting) were reported most commonly (Keating, 2015). Patients treated with Nintedanib who experience diarrhea should maintain hydration and take antidiarrheal therapy as soon as symptoms occur. Dose reduction can also be taken into consideration (Corte et al., 2015).

The timing of antifibrotic therapy may influence the outcome of IPF treatment. A retrospective study indicated that patients who initiated Nintedanib immediately after IPF diagnosis might have reduced hospitalization risk and medical costs compared with those who start treatment later (Singer et al., 2022). A *post hoc* analysis of INPULSIS program suggested that probable UIP with traction bronchiectasis in high-resolution CT might be sufficient for patients to benefit from Nintedanib, implying the importance of early antifibrotic therapy (Lobo, 2016).

Although high-quality clinical trial data are currently lacking to directly demonstrate that Nintedanib is effective in end-stage IPF, the latest *post hoc* analyses of the INPULSIS and INSTAGE programs suggest that it has some effect in IPF patients with more severely impaired gas exchange (Richeldi et al., 2020).

#### 6.1.3 Implications of Pirfenidone and Nintedanib for Anti-Idiopathic Pulmonary Fibrosis Drug Discovery

With the launch of Pirfenidone and Nintedanib in multiple countries, the number of IPF patients not receiving anti-fibrotic therapy will decrease in the future, and it will become increasingly difficult to observe the natural progression of IPF. For ethical reasons and feasibility of patient recruitment, more and more clinical trials of new drugs will use Pirfenidone and Nintedanib as controls. This sets a higher standard for the efficacy of new drugs. In addition, there will be a trend to combine new drugs with existing anti-fibrotic therapies in clinical trials, and whether they can bring additional benefits will be the determining factor. Furthermore, whether a single drug is sufficient to effectively delay the progression of IPF is an important pharmacoeconomic proposition. Since IPF is a rapidly progressive and irreversible disease, whether anti-fibrotic therapy is effective in different stages of IPF, especially in end-stage IPF and suspected IPF, also deserves further study. There are currently no high-quality clinical trials that directly demonstrate the efficacy of antifibrotic therapy in end-stage IPF. In addition, whether patients with suspected IPF on unbiopsied CT images without biopsy require prophylactic antifibrotic therapy remains to be further investigated.

### 6.2 Anti-Idiopathic Pulmonary Fibrosis Drugs Targeting Cytokine and Growth Factor Pathways in Clinical Trials

With the continuous exploration of the pathophysiological process of IPF and the continuous clarification of the cytokine and growth factor pathways, the targets of IPF treatment have also become diversified. Many targets and drugs developed based on them have entered clinical trials (Table 2). Although most of them fail to enter phase III clinical trials, a few drugs show promise in the treatment of IPF. Currently, pamrevlumab (CTGF mAb) and PRM-151 (recombinant pentraxin 2) have successfully entered Phase III clinical trials.

**TABLE 2:** Emerging drugs targeting cytokine and growth factor pathways for IPF treatment in clinical trials.

Targets	Drugs	Clinical trial information
TGF-β	GC1008/fresolimumab (TGF-β antibody)	Phase 1 (completed, NCT00125385)
TGF-β	PLN-74809 (integrin αvβ6/αvβ1 inhibitor)	Phase 2 (recruiting, NCT04072315)
		Phase 2 (recruiting, NCT04396756)
TGF-β	TRK-250 (anti-TGF-β mRNA nucleic acid)	Phase 1 (Active, not recruiting, NCT03727802)
TGF-β	TD139 (suppress TGF-β receptor by targeting galectin-3)	Phase 1/2 (completed, NCT02257177)
		Phase 2 (recruiting, NCT03832946)
TGF-β1, CTGF, IL-β23p19, IL-6	PBI-4050 (inhibitor of TGF-β1, CTGF, IL-β23p19, IL-6)	Phase 2 (completed, NCT02538536)
CTGF	Pamrevlumab (CTGF mAb)	Phase 3 (recruiting, NCT03955146); Phase 3 (recruiting, NCT04419558)
IL-13	QAX576 (IL-13 mAb)	Phase 2 (terminated, NCT01266135)
	Tralokinumab (IL-13 mAb)	Phase 2 (terminated, NCT01629667)
	Lebrikizumab (IL-13 mAb)	Phase 2 (completed, NCT01872689)
IL-4, IL-13	SAR156597 (IL-4 and IL-13 Antibody)	Phase 2 (completed, NCT02345070)
CCL-2	CNTO 888 (CCL2 mAb)	Phase 2 (terminated, NCT00786201)
JNK	Jaktinib Dihydrochloride Monohydrate (JNK1/2 inhibitor)	Phase 2 (recruiting, NCT04312594)
	CC-90001 (JNK1/2 inhibitor)	Phase 2 (active, not recruiting, NCT03142191)
Src	Saracatinib (Src kinase inhibitor)	Phase 1/2 (recruiting, NCT04598919)
Hedgehog pathways	taladegib/ENV-101 (Smo receptor inhibitor)	Phase 2 (not yet recruiting, NCT04968574)
ROCK	KD025 (ROCK2 inhibitor)	Phase 2 (completed, NCT02688647)
Leukotrienes	MN-001/Tipelukast (leukotriene receptor antagonist)	Phase 2 (active, not recruiting, NCT02503657)
LPC-ATX-LPA	BMS-986278 (LPA1R antagonist)	Phase 2 (recruiting, NCT04308681)

### 6.3 Information About Clinical Trials That Target Complications and Comorbidities of Idiopathic Pulmonary Fibrosis

Complications and comorbidities are of substantial significance to the disease progression and quality of life in IPF patients.

Cough affects up to 80% of patients with IPF, is frequently disabling, and lacks effective therapy. A 24-week, double-blind, two-treatment, 24 patients enrolled, two-period crossover trial showed that thalidomide improved cough and respiratory quality of life in patients with IPF (Horton et al., 2012).

Patients with IPF are also at risk of developing pulmonary hypertension as the underlying condition worsens (Tanaka et al., 2017). The BUILD-3 program showed no benefit of Bosentan in the life quality of IPF patients (King et al., 2011a). However, a prospective study showed that Bosentan was associated with a trend toward decreased adverse events and improved respiratory status (Tanaka et al., 2017). Sildenafil is an FDA-approved drug for pulmonary arterial hypertension. A double-blind randomized clinical trial showed that the combination of Sildenafil and Nintedanib significantly reduced BNP (B-type natriuretic peptide, an indicator of heart failure) in IPF patients with right ventricular dysfunction compared with Nintedanib alone, but did not improve HRQL (Behr et al., 2019; Suarez-Cuartin and Molina-Molina, 2019). More recently, a systematic review and meta-analysis of RCTs reported that Sildenafil probably reduced all-cause mortality in IPF patients (Pitre et al., 2022), although more studies are needed to confirm this. The 2015 ATS/ERS/JRS/ALAT clinical practice guideline conditionally recommends against the use of Bosentan and Sildenafil, while the 2011 version strongly recommends against the use of them.

Increased bacterial load and loss of microbial diversity have been reported in IPF (Han et al., 2014; Molyneaux et al., 2014), which are associated with disease progression and immune response (Huang et al., 2017; Molyneaux et al., 2017). However, the role of several antibiotics in IPF appears to be controversial. A retrospective study of 85 AE-IPF patients showed that azithromycin may improve survival in patients with AE-IPF compared with a fluoroquinolone-based regimen (Kawamura et al., 2017). On the contrary, a randomized controlled crossover study of 25 IPF patients does not support the use of low-dose azithromycin for chronic cough in IPF (Guler et al., 2021). In addition, a pragmatic randomized unblinded clinical study of 513 IPF patients didn’t support the additional use of co-trimoxazole or doxycycline (Martinez et al., 2021). Thus, the selection of antibiotics and the effect of them on immune cells need to be further explored.

Gastroesophageal reflux occurs in a high proportion of IPF patients (Raghu et al., 2015), and chronic microaspiration secondary to gastroesophageal reflux is thought to play a role in disease pathogenesis and progression (Savarino et al., 2013). The 2015 ATS/ERS/JRS/ALAT clinical practice guideline conditionally recommends anti-acid therapy. However, whether anti-acid therapy is effective in IPF patients remains highly controversial (Lee et al., 2011; Lee et al., 2013; Kreuter et al., 2016; Raghu et al., 2018; Tran et al., 2021). In addition, whether anti-acid treatment should choose anti-acid drugs or radical therapy (such as Nissen fundoplication) requires further study (Johannson et al., 2017). Therefore, to investigate the efficacy of anti-acid therapy in slowing the progression of IPF, a phase III, randomized, placebo-controlled, double-blind, multicenter clinical trial using lansoprazole is currently enrolling patients with IPF (NCT04965298).

## 7 Challenge and Outlook

Research on the pathogenesis of IPF has made considerable progress. After years of preclinical research and clinical studies, the pathogenesis of IPF has changed from simple inflammation to abnormal epithelial-mesenchymal crosstalk and other pathogenic mechanisms. Based on the emerging pathological mechanism, many studies have systematically studied the key roles of cytokine and growth factor pathways in the pathogenesis of IPF. The approval of Pirfenidone and Nintedanib shows that therapy targeting these pathways is very promising. Although investigational drugs targeting some cytokines such as IFN-γ, TNF-α, and IL-13 have not shown satisfactory efficacy, the development of anti-IPF drugs targeting cytokine and growth factor pathways is still promising. Currently, there are numerous clinical trials investigating the efficacy of these drugs in the treatment of IPF and more drugs may be approved based on the results of these trials.

Advances in the field of cytokine and growth factor pathways would help slow disease progression, prolong lives, and improve the quality of life of IPF patients in the future. Therefore, this review tried to provide an overview of cytokines, growth factors, and their signaling pathways in IPF. Furthermore, key information about emerging drugs targeting these pathways for IPF treatment in clinical trials is provided.

Based on the findings of this review, the following points need to be considered in future studies to make strategies targeting these signaling pathways more promising for the treatment of IPF patients. Although upregulating or antagonizing certain cytokine or growth factor signaling pathways alone may not reverse differentiated cells, promoting fibrotic phenotype transition is more promising. Since cytokine and growth factor signaling pathways also play vital roles in other physiological and pathological processes, including damage repair, defense against infection, autoimmune disease, and antitumor immunity, long-term non-selective targeting of these pathways may lead to undesired side effects. Therefore, the identification of more specific targets and the development of drugs having high selectivity on them, the optimization of drug delivery to increase drug concentrations at the target site, and the identification of biomarkers to help select patients who might benefit more from drugs are all worth exploring. In addition, combination therapy with Pirfenidone and Nintedanib might bring better clinical benefits for IPF patients than single drug administration.

One of the novelties of this review is giving an extensive description of the multiple roles of cytokine and growth factor pathways in pulmonary fibrosis, which provide new insights into therapeutic strategies for IPF. These pathways form a complex network in the pathological process of pulmonary fibrosis, and exploring the crosstalk between different signaling cascades may help discover more potential targets. Clinical trials are a critical stage that all approved drugs must go through. Some of the drugs in clinical trials for the treatment of IPF target cytokines and growth factors. Therefore, we summarize key information from these clinical trials and hope to give insights for the development of more effective drug.

Although IPF is idiopathic by definition, it may become less mysterious with progress in the understanding of the pathogenesis of IPF. This review showed that the number of therapeutic targets from cytokine or growth factor signaling pathways and new targeted drugs entering clinical trials is increasing. These advances show that slowing disease progression, prolonging prognosis, and improving quality of life might be possible in the future for patients with IPF.

In conclusion, cytokine and growth factor signaling pathways are indispensable in the pathogenesis of IPF, and this review hopes to provide theoretical support for the development of novel anti-pulmonary fibrosis drugs targeting cytokine and growth factor pathways.

## Glossary

AECs: alveolar epithelial cells
AE-IPF: acute exacerbation of idiopathic pulmonary fibrosis
AT I/AEC I: type I AECs
AT II/AEC II: type II AECs
ATX: autotaxin
BALF: bronchoalveolar lavage fluid
BLM: bleomycin
COVID-19: corona virus disease 2019
COX: cyclooxygenase
CTGF: connective tissue growth factor factors
DC: dendritic cell
Th: helper T cell
DLco/VA: diffusion capacity for carbon monoxide per liter of alveolar volume
ECM: extracellular matrix
EGF: epidermal growth factor
EMT: epithelial-mesenchymal transition
EndoMT: endothelial-to-mesenchymal transition
ER: endoplasmic reticulum
ERK: extracellular signal-regulated kinase
ERS: endoplasmic reticulum stress
FAK: focal adhesion kinase
FEV1: forced expiratory volume in one second
FGF: fibroblast growth factor
FVC: forced vital capacity
GF: growth factor
Hh: Hedgehog
HLF-1: human lung fibroblast-1
HRQL: health-related quality of life
IGF: insulin-like growth factor
IL: interleukin
IPF: idiopathic pulmonary fibrosis
JAK/STAT: Janus kinase/signal transducer and activator of transcription
JNK: c-Jun N-terminal kinase
LAP: Latency-associated peptide
LPA: Lysophosphatidic acid
LT: Leukotriene
LTBP: Latent TGF-β binding protein
miR: micro-RNA
MAPK: mitogen-activated protein kinase
MKK: Mitogen-activated protein kinase kinase
MMP/TIMP: matrix metalloproteinase/tissue inhibitor of metalloproteinase
mAb: monoclonal antibody
MSC: Mesenchymal stem cell
mtROS: mitochondrial reactive oxygen species
PAI: plasminogen activation inhibitors
PDGF: platelet-derived growth factor
PI3K/Akt/mTOR: phosphatidylinositol 3-kinase/protein kinase B/mammalian target of rapamycin
PLA2: phospholipase A2
PPAR-γ: peroxisome proliferators-activated receptors γ
Rho/ROCK: Rho/Rho-associated coiled-coil kinase
RCT: randomized controlled trial
ROS: reactive oxygen species
SFK: Src family kinases
SPHK1/S1P/S1PR: sphingosine kinase/sphingosine 1 phosphate/sphingosine 1 phosphate receptor
TBR: TGF-β receptor
TF: tissue factor
TGF-β: transforming growth factor-β
TIMPs: tissue inhibitors of metalloproteinases
UPR: unfolded protein response
VEGF: vascular endothelial growth factor
Wnt: Wingless/Integrated
α-SMA: α-smooth muscle actin
